# *Haimormus shimojiensis*, a new genus and species of Pseudozeuxidae (Crustacea: Tanaidacea) from a submarine limestone cave in Northwestern Pacific

**DOI:** 10.7717/peerj.4720

**Published:** 2018-04-26

**Authors:** Keiichi Kakui, Yoshihisa Fujita

**Affiliations:** 1 Faculty of Science, Hokkaido University, Sapporo, Hokkaido, Japan; 2 Okinawa Prefectural University of Arts, Naha, Okinawa, Japan

**Keywords:** Malacostraca, Peracarida, Marine cave, Ryukyu Islands, Key, Japan

## Abstract

We establish a new pseudozeuxid genus *Haimormus* gen. nov. based on a new species *Haimormus shimojiensis* sp. nov. which was collected from a submarine limestone cave with the entrance at 35 m depth, in the Northwestern Pacific Ocean. *H. shimojiensis* differs from the other confamilial members, *Pseudozeuxo belizensis* Sieg, 1982 and *Charbeitanais spongicola* Bamber & Bird, 1997, in having the pleonite 1 without the pleopod, the pereopods 2 and 3 propodus with a ventral spiniform seta, and the pereopods 4–6 propodus with one long and two short dorsodistal setae. A key to females of species of Pseudozeuxidae is presented. This is the first tanaidacean report from submarine caves around Japan.

## Introduction

**Figure 1:**
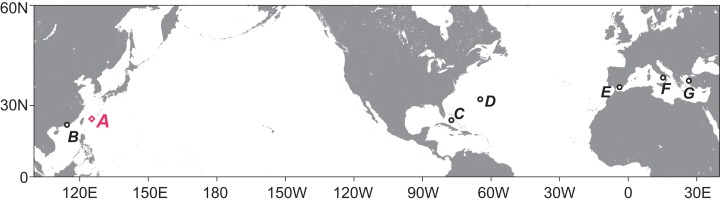
Map showing the regions with reports of tanaidaceans inhabiting submarine caves. (A) Sampling site for this study, Shimoji-jima Island. (B) Hong Kong (four species). (C) Bahamas (two species). (D) Bermuda (two species). (E) South of Iberian Peninsula (six species). (F) Capo Palinuro (one species). (G) Lesvos Island (one species). The map and plots were generated with GMT5 software (Wessel et al., 2013).

Tanaidacean crustaceans are typically benthic, small animals, up to a few millimeters long. They are recorded from almost all types of marine habitat (Blazewicz-Paszkowycz, Bamber & Anderson, 2012), and the number of living species is approximately 1,400 (Anderson, 2017). One of the most extreme habitats they utilize is undersea caves that have underwater entrance(s) open to the surrounding marine environment (hereafter we call such caves “submarine caves”). Submarine caves are known as an interesting habitat harboring cave-endemic species (Okanishi & Fujita, 2018), “living fossils” (Kase & Hayami, 1992; Johnson et al., 2012), and deep-sea creatures (Vacelet & Boury-Esnault, 1995), but are relatively unstudied because specialized cave-diving equipment and techniques are essential to investigate such areas. To date, submarine-cave tanaidaceans have been reported from six regions in the Northern Hemisphere (Fig. 1), and 16 species in 14 genera (including one taxon identified at the genus level) have been recorded (Table 1) (Băcescu, 1980; Guţu & Iliffe, 1985, 2001, 2008; Bamber, 2000; Bird & Bamber, 2000; Bamber & Sheader, 2003; Akoumianaki & Hughes, 2004; Bamber, Evans & Robbins, 2008; Navarro-Barranco et al., 2013, 2015; Gerovasileiou et al., 2016; García-Herrero et al., 2018). From the Pacific Ocean, four species have been reported so far: *Paradoxapseudes mortoni* (Bamber, 1997) in Apseudidae; *Tanapseudes ormuzana* Băcescu, 1978 in Kalliapseudidae; *Unguispinosus hodgsoni* (Bamber, 2000) in Parapseudidae; and *Paratanais clarkae* Bird & Bamber, 2000 in Paratanaidae.

**Table 1:** Tanaidaceans recorded from undersea caves with underwater entrance(s) opening to the surrounding marine environment.

Superfamily/family	Species	Selected source	Region in Fig. 1
**Apseudoidea**			
Apseudidae	*Apseudes* sp.	Navarro-Barranco et al. (2013)	E
	*Apseudopsis latreilli*	Navarro-Barranco et al. (2013)	E
	*Paradoxapseudes bermudeus*	Guţu & Iliffe (1985)	D
	*Paradoxapseudes intermedius*	Gerovasileiou et al. (2016)	G
	*Paradoxapseudes mortoni*	Bamber (2000)	B
Kalliapseudidae	*Tanapseudes ormuzana*	Bamber & Sheader (2003)	B
Metapseudidae	*Hoplopolemius propinquus*	Guţu & Iliffe (1985)	D
Parapseudidae	*Unguispinosus hodgsoni*	Bamber & Sheader (2003)	B
	*Swireapseudes birdi*	Guţu & Iliffe (2008)	C
**Paratanaoidea**			
Leptocheliidae	*Chondrochelia savignyi*	Akoumianaki & Hughes (2004)	F
	*Grallatotanais antipai*	Guţu & Iliffe (2001)	C
Paratanaidae	*Paratanais clarkae*	Bird & Bamber (2000)	B
Pseudotanaidae	*Pseudotanais* (*P.*) *isabelae*	García-Herrero et al. (2018)	E
Pseudozeuxidae	*Haimormus shimojiensis* sp. nov.	This study	A
Tanaellidae	*Araphura brevimanus*	García-Herrero et al. (2018)	E
**Tanaidoidea**			
Tanaididae	*Tanais dulongii*	Navarro-Barranco et al. (2015)	E
	*Zeuxo coralensis*	Navarro-Barranco et al. (2015)	E

Recently, the second author and colleagues have actively investigated the invertebrate fauna in submarine caves around the Ryukyu Islands, southwestern Japan. Their survey has resulted in many discoveries in crustacean taxonomy such as: a previously unknown species of the order Bochusacea (*Thetispelecaris kumejimensis* Shimomura, Fujita & Naruse, 2012), the first record of the order from submarine caves in the Pacific Ocean; a previously unknown species of the order Thermosbaenacea (*Halosbaena okinawaensis* Shimomura & Fujita, 2017b); a previously unknown alpheid shrimp species in a new genus (*Caligoneus cavernicola* Komai & Fujita, 2018); two previously unknown crab species (*Catoptrus iejima* Fujita & Naruse, 2011; *Lipkemera iejima* Naruse & Fujita, 2015); and the first female of the cavenicolous mysid (*Heteromysoides simplex* Hanamura & Kase, 2001) (Fujita & Naruse, 2011; Shimomura, Fujita & Naruse, 2012; Naruse & Fujita, 2015; Shimomura & Fujita, 2017a, 2017b; Komai & Fujita, 2018). Tanaidaceans were also obtained during the surveys, one species of which is studied in this paper, which belongs to Pseudozeuxidae, but could not be assigned to any known genera. We thus describe the species as new and establish a new genus for it. A key to species of Pseudozeuxidae is also provided. This is the first report of tanaidaceans from submarine caves around Japan.

## Materials and Methods

All tanaidaceans were collected by a scuba diving from a submarine limestone cave “Akuma-no-Yakata (Devil’s Hole)” on September 1, 2017. This cave is located on a reef slope at Shimoji-jima Island, Miyako Island Group, southwestern Japan (24°49′22.51″N, 125°08′07.84″E; Fig. 1A), and its entrance lies at a depth of about 35 m; detailed information of the cave is included in Anker & Fujita (2014). From the inside of the cave, one brittle-star and more than 14 decapod species have been found so far (Fujita, Naruse & Yamada, 2013; Anker & Fujita, 2014; Okanishi & Fujita, 2018). Mud deposited in holes or gaps of the cave wall was collected by using a “yabby pump (commercial suction pump)”, from a totally dark point at 18 m depth, ca. 80 m from the cave entrance. Tanaidaceans were sorted from the mud sample, and fixed and preserved in 99% ethanol. The studied specimens were deposited in the crustacean collection of the National Museum of Nature and Science, Tsukuba (NSMT), Japan and in the University Museum Fujukan, University of the Ryukyus (RUMF), Japan. The methods used for dissection, preparation of slides, light microscopy, scanning electron microscopy (SEM), and drawing were as described by Kakui & Angsupanich (2012). DNA extraction was attempted for one paratype specimen (NSMT-Cr 26837), but subsequent PCR was not successful.

**Figure 2:**
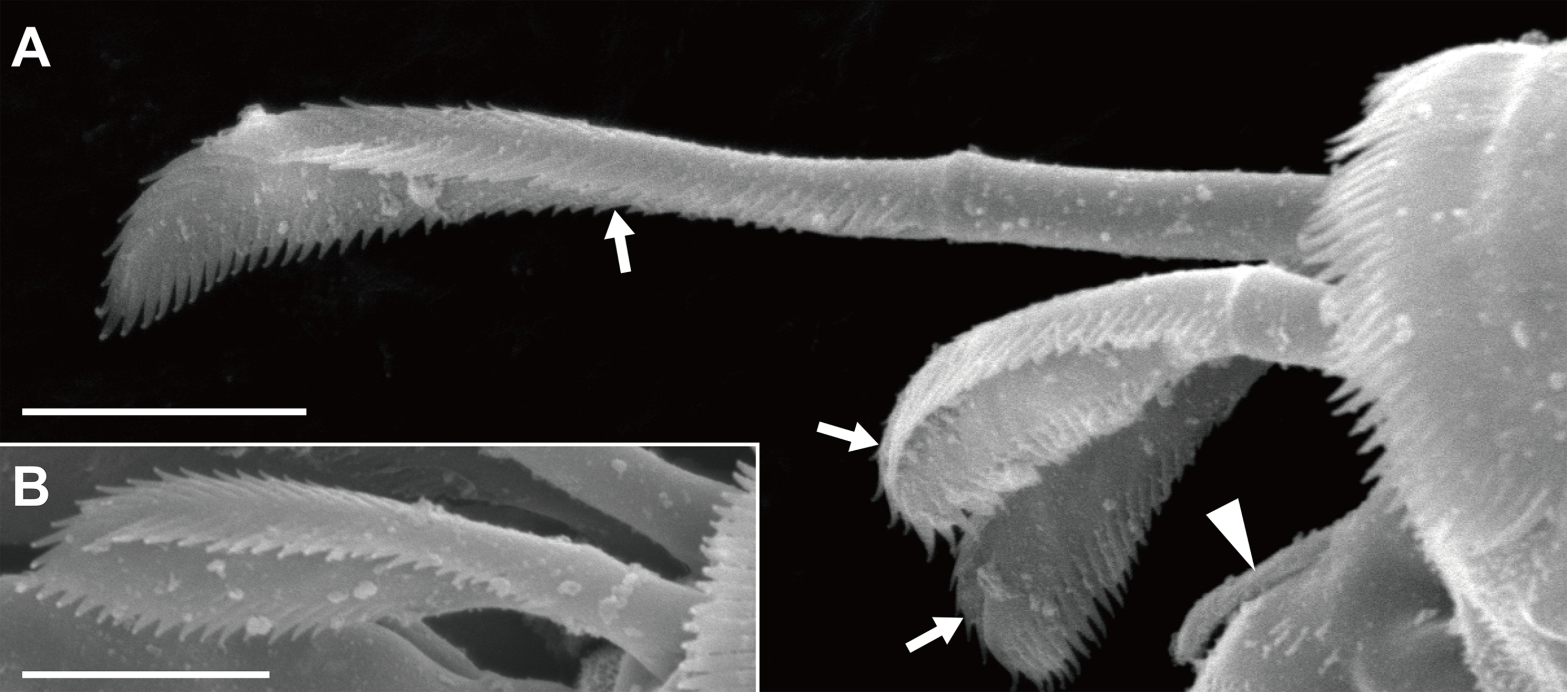
*Haimormus shimojiensis* gen. et sp. nov., paratype female (NSMT-Cr 26837), SEM images. (A) Subdistal region of right pereopod 5, outer view; arrowhead and arrows indicate dorsoproximal seta on dactylus and triserrate setae, respectively. (B) Triserrate seta on right pereopod 4, outer view. Scale: 0.005 mm.

Orientation and terminology here follow Larsen (2003), except that the term “plumose sensory seta(e)” (PSS; Bird, 2011) is used instead of “broom seta(e).” We proposed one setal term, “triserrate seta(e),” for the setae found in the dorsodistal region of the pereopods 4–6 propodus; the seta expands distally and bears three (dorsal, ventral, and outer/inner) rows of serrations (Fig. 2). Body length (BL) was measured from the tip of the eyelobe to the tip of the pleotelson, and body width at the widest portion of the carapace (CW, carapace width). Appendages were measured only in the holotype specimen. Measurements were made axially: dorsally on the body, antennules, antennae, and uropods; laterally on the pereopods. The suffixes in the newly proposed Japanese names, viz., “-ka” and “-zoku” represent the taxonomic ranks family and genus, respectively, in the Japanese language.

The electronic version of this article in portable document format will represent a published work according to the International Commission on Zoological Nomenclature (ICZN), and hence the new names contained in the electronic version are effectively published under that Code from the electronic edition alone. This published work and the nomenclatural acts it contains have been registered in ZooBank, the online registration system for the ICZN. The ZooBank LSIDs (Life Science Identifiers) can be resolved and the associated information viewed through any standard web browser by appending the LSID to the prefix http://zoobank.org/. The LSID for this publication is: urn:lsid:zoobank.org:pub:3019D534-3E50-49D3-AC6E-8EB5A80DD1BB. The online version of this work is archived and available from the following digital repositories: PeerJ, PubMed Central, and CLOCKSS.

### Systematics

**Family Pseudozeuxidae Sieg, 1982**
[New Japanese name: Tijimi-tanaisu-ka]

**Diagnosis, female** (amended from Bird & Larsen, 2009). Eyes present. Pereonite 1 far longer than width of pereopod-1 coxa. Pleon narrower than pereonite 6, consisting of pleonites 1–5 and pleotelson, with combined length of pleonites 1–5 shorter than pereonite 6. Antennule with three articles. Antenna with six articles; article 2 with one or two dorsal spiniform setae; article 3 with dorsal spiniform seta. Mandibular molar well developed, with broad masticatory region; right mandibular incisor with subdistal crenulation. Labium with two pairs of lobes. Maxillipedal bases fused or not fused medially; endites not fused medially. Cheliped attachment via triangular sclerite. Pereopod coxa present on pereopods 1–3 but absent on pereopods 4–6; pereopods 4–6 dactylus–unguis claw-shaped. Pleopod 1 present or absent; pleopods 2–5 absent. Uropodal endopod with one or two (occasionally three) articles; exopod with one or two articles.

**Diagnosis, male** (modified from Bird & Larsen, 2009). Body not compressed but shorter than in female and with more elongate cephalothorax. Antennule with three articles. Functional mouthparts retained.

**Genera included.** *Pseudozeuxo* Sieg, 1982 (one species); *Charbeitanais* Bamber & Bird, 1997 (one species); *Haimormus* **gen. nov.** (one species).

**Genus *Haimormus* gen. nov.**
[New Japanese name: Ashinashi-tijimi-tanaisu-zoku]
urn:lsid:zoobank.org:act:5C5EEDB4-A9C3-406C-AA25-9D83846569AD

**Type species.** *Haimormus shimojiensis* **sp. nov.**, by original designation.

**Diagnosis, female.** Maxillipeds with bases not fused medially; endite with long simple seta and three short spiniform setae in ventrodistal region, and two long and one short spiniform setae in inner dorsodistal region. Pereopods 2 and 3 propodus with ventral spiniform seta. Pereopods 4–6 propodus with one long and two short dorsodistal setae. Pleopod 1 absent. Uropodal exopod uniarticulate.

Male unknown.

**Etymology.** The genus name (masculine) is named in honor of Michitaka Shimomura who greatly contributes to the taxonomy of tiny crustaceans from submarine caves around Japan in recent years (an anagram of his last name).

**Figure 3:**
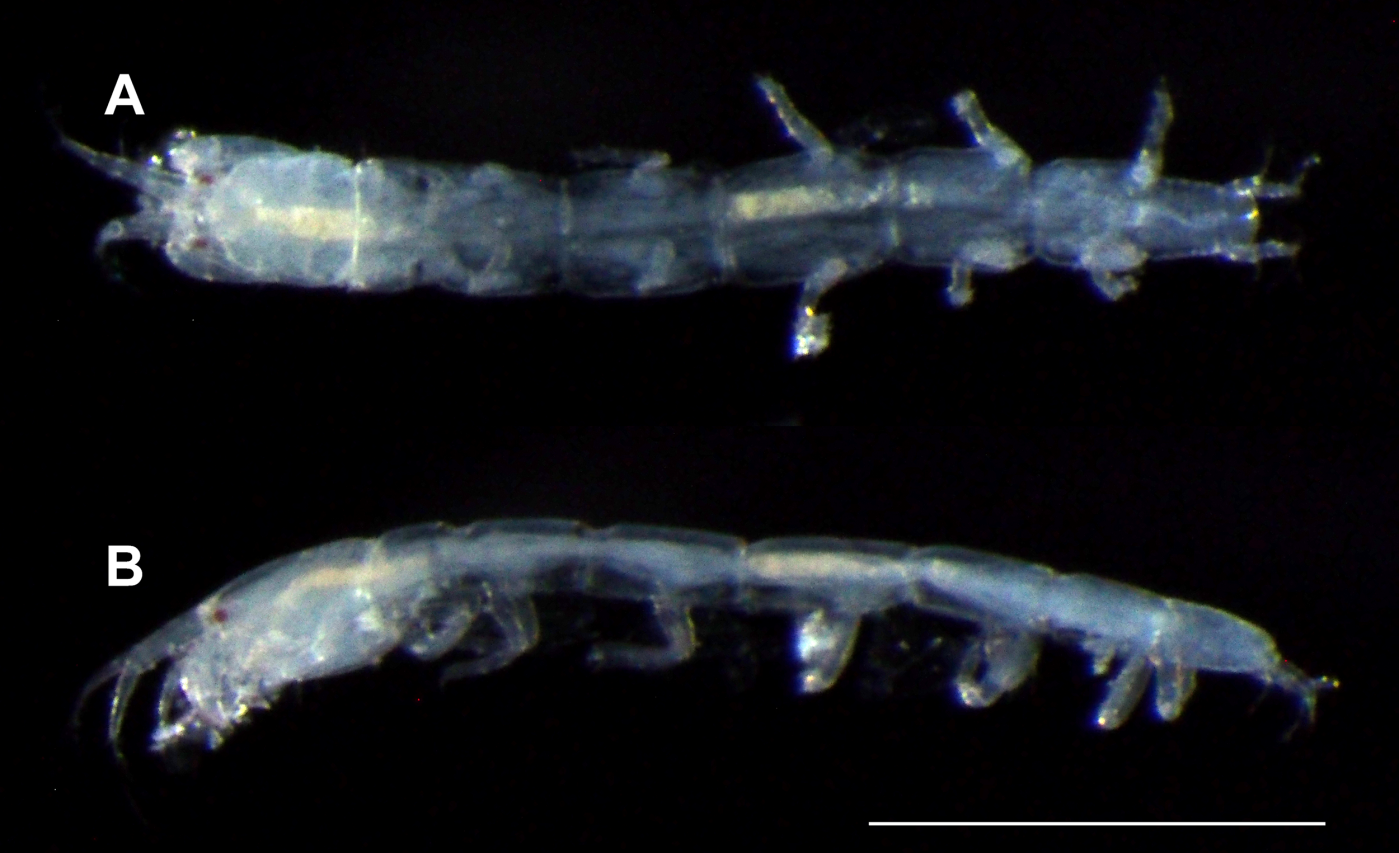
*Haimormus shimojiensis* gen. et sp. nov., paratype female (RUMF-ZC-6004), fixed specimen. (A) Body, dorsal view. (B) Body, left view. Scale: 0.5 mm.

**Figure 4:**
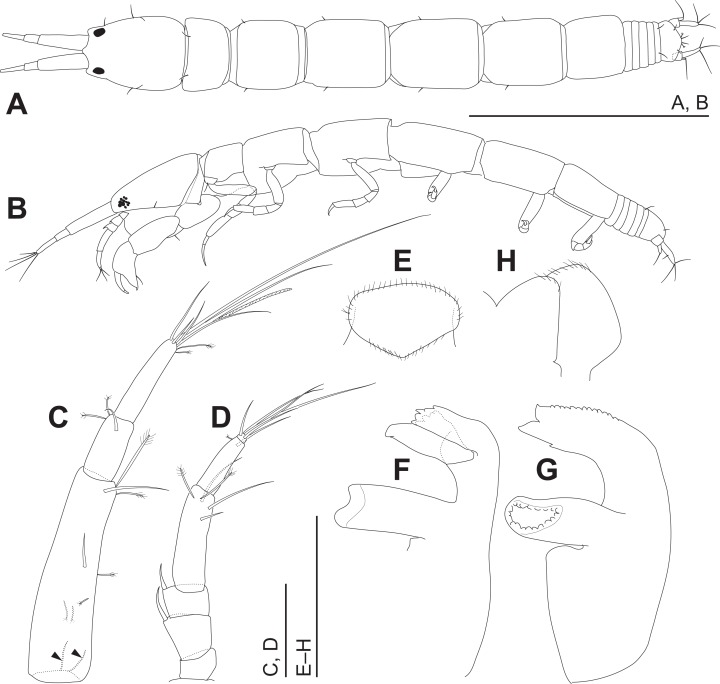
*Haimormus shimojiensis* gen. et sp. nov., holotype female. (A) Body, dorsal view. (B) Body, left view. (C) Right antennule. (D) Right antenna. (E) Labrum, ventral view. (F) Left mandible. (G) Right mandible. (H) Labium, ventral view. Scale: 0.5 mm for (A, B); 0.05 mm for the others.

**Figure 5:**
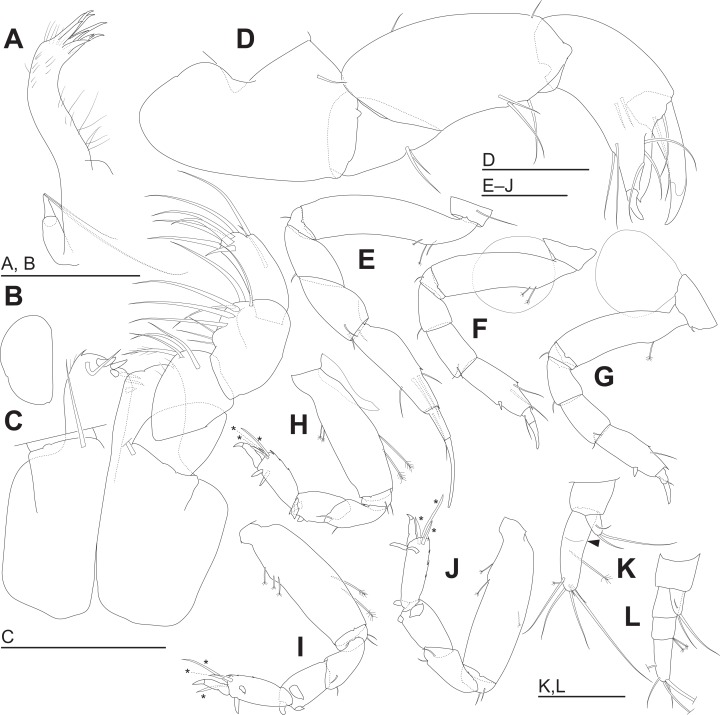
*Haimormus shimojiensis* gen. et sp. nov. (A–K) holotype female; (L) paratype female (RUMF-ZC-6004). (A) Left maxillule. (B) Right maxilla. (C) Maxillipeds, ventral view, right palp and setal ornamentation on left endite omitted. (D) Right cheliped, outer view. (E–J) Right pereopods 1–6, outer views. (K) Right uropod, outer dorsal view, with arrowhead indicating pseudoarticulation on endopod. (L) Right uropod, outer dorsal view. *, triserrate seta. Scale: 0.05 mm.

**Figure 6:**
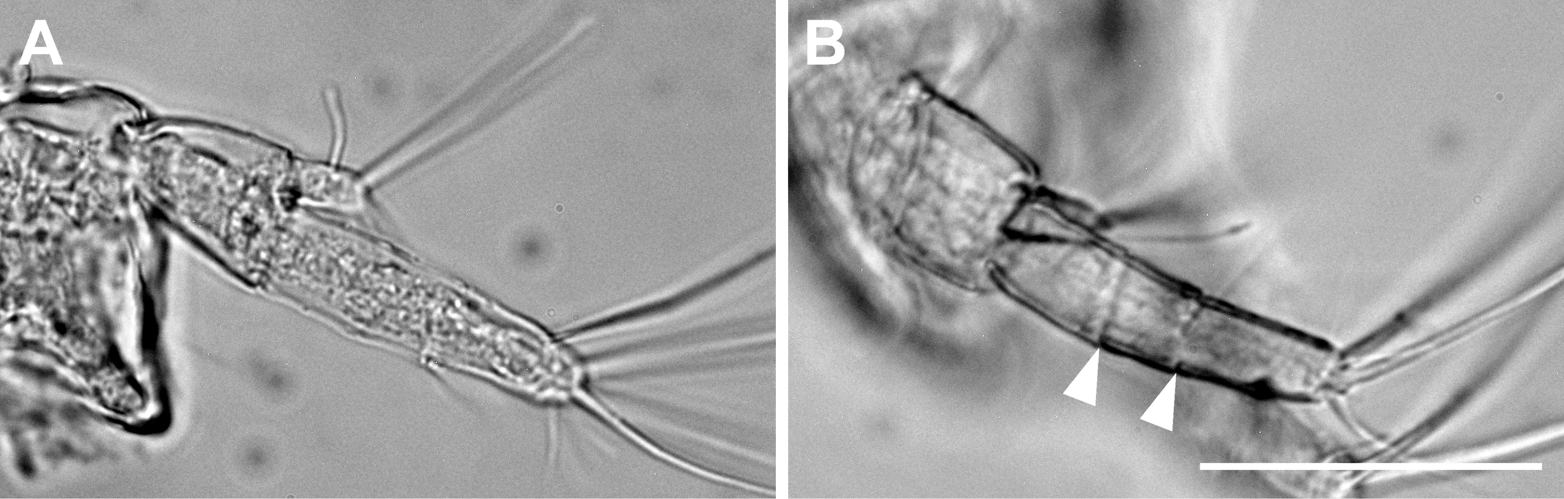
*Haimormus shimojiensis* gen. et sp. nov. (A) Uniarticulate uropodal endopod (holotype, NSMT-Cr 26836), dorsal view. (B) Triarticulate uropodal endopod (RUMF-ZC-6004), outer view, with arrowheads indicating articulations. Scale: 0.05 mm.

***Haimormus shimojiensis* sp. nov.**
[New Japanese name: Shimoji-tijimi-tanaisu]
urn:lsid:zoobank.org:act:916C8C7C-5C81-43AF-9694-E7E7A372FA0C
Figures 2–6

**Diagnosis.** Same as for the genus.

**Etymology.** The specific name is an adjective referring to the type locality.

**Material examined.** Holotype. Female with developing oostegites, NSMT-Cr 26836 (BL 1.21 mm, CW 0.16 mm), six slides and one vial; “Akuma-no-Yakata (Devil’s Hole),” Shimoji-jima Island, Miyako Island Group, southwestern Japan, Northwestern Pacific Ocean (24°49′22.51″N, 125°08′07.84″E), 18 m depth, mud, 1.ix.2017, collected by Y. Fujita. Paratypes. Female, NSMT-Cr 26837 (BL 1.04 mm, CW 0.14 mm), one SEM stub, one slide and one vial; female with developed oostegites, RUMF-ZC-6004 (BL 1.17 mm, CW 0.14 mm), three slides and one vial; same locality as holotype.

**Description of female.** Based on holotype.

Body (Figs. 3, 4A and 4B) dorsoventrally flattened, 7.56 times as long as CW, translucent in ethanol (Fig. 3); body wall not heavily calcified. Cephalothorax 0.16 times as long as BL, 1.27 times as long as wide, acorn-shaped in dorsal view, with two pairs of lateral simple setae (one seta lost); rostrum triangular; eye present. Pereonites 1–6 with length ratio of 1.00:1.49:1.92:2.04:1.86:1.45; pereonites 1, 2, 6 wider than long, pereonites 3–5 longer than wide; pereonite 1 with pair of dorsolateral simple setae; pereonites 2–5 with pair of lateral simple setae. Pleon 0.10 times as long as BL. Pleonites narrower than pereonite 6; all wider than long, similar in shape, but the width gradually narrower from pleonites 1–5; pleonite 5 with pair of dorsolateral simple setae. Pleotelson 0.56 times as long as wide, almost same width to pleonite 4, pentangular in dorsal view, with pair of subdistal simple setae, pair of distal simple setae and pair of distal PSS.

Antennule (Fig. 4C) as long as cephalothorax; articles 1–3 with length ratio of 1.00:0.31:0.48. Article 1 with one middle and one distal simple setae, and several PSS; several fine setae on proximal margin (Fig. 4C, arrowheads). Article 2 with distal simple seta and two distal PSS. Article 3 with six distal simple setae, two distal PSS, and distal aesthetasc. Antenna (Fig. 4D) 0.72 times as long as antennule; articles 1–6 with length ratio of 1.00:1.97:1.22:4.64:2.85:0.31. Article 1 naked. Articles 2 and 3 each with dorsodistal spiniform seta. Article 4 with one subdistal and two distal simple setae and two distal PSS. Article 5 with distal simple seta and PSS. Article 6 with five distal simple setae.

Labrum (Fig. 4E) not projected anteriorly, setulate. Mandibles (Figs. 4F and 4G) with molar well developed; masticatory region broad. Incisor of left mandible (Fig. 4F) with several teeth; lacinia mobilis with several small and one large teeth. Incisor of right mandible (Fig. 4G) bifurcate distally, with subdistal anterior crenulation and subdistal posterior process. Labium (Fig. 4H) bilobed; inner and outer lobes setulate. Maxillule (Fig. 5A) with endite bearing eight distal single-tipped and one distal bifurcate spines and fine setules; palp biarticulate, with two distal simple setae. Maxilla (Fig. 5B) oval, naked. Maxillipeds (Fig. 5C) with bases not fused medially, each bearing simple seta at insertion of palp. Endites not fused medially, reaching beyond distal margin of palp article 1, each with long simple outer seta and three short (one inner most one relatively shorter and more blunt than other two) spiniform setae in ventrodistal region, and two long and one short spiniform setae in inner dorsodistal region; outer margin setulate. Palp article 1 naked; article 2 with several fine setae, three distal simple setae, and distal bipinnate seta in inner region; article 3 with three proximal fine setae and six simple setae in inner region; article 4 with one mid-dorsal and seven distal simple setae. Epignath lost during dissection.

Cheliped (Fig. 5D) with triangular articulation with cephalothorax via sclerite. Basis longer than wide, with free posterior portion just shorter than anterior, latter with dorsal simple seta. Merus with two ventral simple setae. Carpus slender, more than twice as long as wide, with one mid-dorsal, one dorsodistal, and three ventral simple setae. Chela as long as carpus, slender, 2.5 times as long as wide; propodal palm longer than fixed finger, with one outer and two inner simple setae at insertion of dactylus; fixed finger with two simple setae on ventral margin, three outer simple setae on cutting surface, and pointed claw; dactylus–unguis slightly longer than fixed finger, with inner proximal simple seta; unguis pointed.

Pereopods 1–6 cylindrical, with length ratio of 1.00:0.66:0.62:0.61:0.68:0.71. Pereopod 1 (Fig. 5E) 0.24 times as long as BL, with length ratio of basis, ischium, merus, carpus, propodus, and dactylus–unguis 1.0:0.1:0.3:0.4:0.6:0.6. Coxa with simple seta. Basis cylindrical, narrow (4.46 times as long as wide), with mid-dorsal simple seta and two mid-dorsal PSS. Ischium with ventral simple seta. Merus with ventrodistal simple seta. Carpus with three ventrodistal simple setae. Propodus with one mid-dorsal, one mid-ventral, and two mid-inner simple setae. Dactylus with proximal simple seta. Unguis twice as long as dactylus, naked. Pereopod 2 (Fig. 5F) with length ratio of articles from basis to dactylus–unguis 1.0:0.1:0.3:0.4:0.5:0.3. Coxa with simple seta (not illustrated; confirmed in left pereopod 2). Basis with two mid-dorsal PSS. Ischium with ventral simple seta. Merus with ventrodistal simple seta. Carpus with two ventrodistal simple setae. Propodus with one mid-dorsal and one mid-inner simple setae, and mid-ventral spiniform seta. Dactylus with proximal simple seta. Unguis 1.7 times as long as dactylus, naked. Pereopod 3 (Fig. 5G) with length ratio of articles from basis to dactylus–unguis 1.0:0.1:0.3:0.4:0.6:0.3; similar to pereopod 2, except basis with one mid-dorsal PSS. Pereopod 4 (Fig. 5H) without coxa. Length ratio of articles from basis to dactylus–unguis 1.0:0.1:0.3:0.3:0.4:0.3. Basis cylindrical (2.89 times as long as wide), with two mid-dorsal and two mid-ventral PSS. Ischium with two ventral simple setae. Merus with two ventrodistal simple setae. Carpus with three distal spiniform setae. Propodus with dorsal serration, two ventrosubdistal spiniform setae, and one long and two short dorsodistal triserrate setae (* in Fig. 5H; cf. Fig. 2). Dactylus with dorsoproximal seta (cf. Fig. 2A, arrowhead). Unguis 0.42 times as long as dactylus, naked. Pereopod 5 (Fig. 5I) with length ratio of articles from basis to dactylus–unguis 1.0:0.1:0.3:0.3:0.4:0.3; similar to pereopod 4 except basis with three mid-dorsal PSS. Pereopod 6 (Fig. 5J) with length ratio of articles from basis to dactylus–unguis 1.0:0.1:0.3:0.3:0.4:0.2; similar to pereopod 4 except basis with one mid-ventral PSS.

Pleopods absent.

Uropod (Fig. 5K) with basal article naked. Endopod 2.60 times as long as basal article, uniarticulate; length/width ratio 3.10; pseudoarticulation present (Fig. 5K, arrowhead); with one subdistal and five distal simple setae and one middle and one distal PSS. Exopod uniarticulate, 0.25 times as long as endopod with one middle and two distal simple setae.

**Variation.** In addition to the holotype (NSMT-Cr 26836), two paratype specimens (NSMT-Cr 26837, RUMF-ZC-6004) were observed. All specimens lacked the pleopod 1. The numbers of simple setae, spiniform setae, triserrate setae, and aesthetascs on the antennule, antenna, cheliped, pereopods, and uropod were identical among the three specimens (pereopod 1 was not observed in NSMT-Cr 26837) with the following exceptions. (1) In the antennule, the article 2 has one (NSMT-Cr 26836) or two (NSMT-Cr 26837, RUMF-ZC-6004) simple setae, and the article 4 has six (NSMT-Cr 26836, RUMF-ZC-6004) or four (NSMT-Cr 26837) distal simple seta. (2) The right antenna of one paratype (RUMF-ZC-6004) has two dorsodistal spiniform setae on the article 2 but no setae on the article 3; the articles 2 and 3 in the other five antennae we observed (i.e., both of NSMT-Cr 26836, both of NSMT-Cr 26837, and left of RUMF-ZC-6004) each has one dorsodistal spiniform seta. (3) The uropodal endopod has five (NSMT-Cr 26836) or four (NSMT-Cr 26837, RUMF-ZC-6004) distal simple setae.

The uropodal endopod has one article in the holotype and one paratype (NSMT-Cr 26837), but it consists of three articles in another paratype (Figs. 5K, 5L and 6).

**Distribution.** So far known only from the type locality.

## Discussion

The reduced pleon, characterized by the pleonites narrower than the pereonite 6 and the combined length of pleonites 1–5 shorter than the pereonite 6, is one of diagnostic characteristics of Pseudozeuxidae found in both sexes. *Haimormus* gen. nov. shares the above character state with the other confamilial genera (*Pseudozeuxo* Sieg, 1982 and *Charbeitanais* Bamber & Bird, 1997), but differs in lacking the pleopod 1 and having the propodus of pereopods 2 and 3 with one ventral spiniform seta (Sieg, 1982; Bamber & Bird, 1997). As no males of *H. shimojiensis* sp. nov. were found in the sample, the degree of sexual dimorphism in antennules, chelipeds, and maxillipeds could not be checked in this genus.

We found a curious morphological difference in the uropod among three observed specimens. Two specimens (holotype and one paratype) have a uniarticulate uropodal endopod but another has a triarticulate one (Figs. 5K, 5L and 6). Although we cannot reject the possibility that the latter is not conspecific to the other two specimens, we consider that, at this moment, all three must be conspecific as the other morphological features are shared among them. Such intraspecific variation in the article number of the uropodal endopod has been reported between sexes in some tanaidaceans (e.g., *Siphonolabrum fastigatum* Sieg, 1986 (Sieg, 1986); *Paratanais tara* Bird, 2011 (Bird, 2011)), but as the case among females in Paratanaoidea (except Leptocheliidae), this is the first case for us. The female uropodal endopod with three or more articles is hitherto restricted in Leptocheliidae and Heterotanoididae (Bird & Larsen, 2009; Bird, 2012). Although *Pseudozeuxo* and *Charbeitanais* do not share this character state (in the two genera, the uropodal endopod is uni- or biarticulate), Bird & Larsen (2009), who illustrated phylogenetic relationships within Paratanaoidea with a parsimonious method using more than 100 morphological characters, suggested the close relation of Pseudozeuxidae to Leptocheliidae and Heterotanoididae (Pseudozeuxidae was nested within the Leptocheliidae–Heterotanoididae clade in their strict consensus tree; Bird & Larsen, 2009). The triarticulate state found in *H. shimojiensis* may support the above hypothetical phylogenetic relationship, and it should be tested by molecular phylogenetic approaches in the future.

García-Herrero et al. (2018) summarized the ecological information of tanaidaceans with reports from marine subterranean environments (e.g., submarine caves and anchialine pools), and showed that most species were not restricted to such environments, i.e., they have reports from open marine environments. In the Pacific, three of four species with report(s) from submarine caves were also collected from the outside of caves (Bamber, 1997; García-Herrero et al., 2018). This study reported *H. shimojiensis* sp. nov. as the fifth tanaidacean species from submarine caves in Pacific. But, as this species lacks remarkable troglomorphic features in the external morphology (such as the elongation of locomotory and sensory appendages; cf. García-Herrero et al., 2018), this species may also be found in open marine environments.

### Key to females of species of Pseudozeuxidae

1. Pleopod 1 absent; pereopods 2 and 3 propodus with ventral spiniform seta *Haimormus shimojiensis* **gen. et sp. nov.**
  – Pleopod 1 present; pereopods 2 and 3 propodus without ventral spiniform seta2
2. Antennal article 2 with two dorsal spinifrom setae; maxillipedal bases not fused; chelipedal carpus with three ventral simple setae; uropodal exopod biarticulatePseudozeuxo belizensis
  – Antennal article 2 with dorsal spiniform seta; maxillipedal bases fused; chelipedal carpus with two ventral simple setae; uropodal exopod uniarticulateCharbeitanais spongicola

## Conclusion

We establish a new pseudozeuxid genus, *Haimormus*, based on the new species *H. shimojiensis* which was collected from a submarine limestone cave in the Northwestern Pacific. *H. shimojiensis* is the first pseudozeuxid lacking the pleopod 1, and this feature easily distinguishes the species from the other two confamilial members. This is the first tanaidacean report from submarine caves around Japan, and just a first step to reveal tanaidacean diversity there. We still have specimens not yet examined, and there are numerous unstudied submarine caves in Japanese waters. Further investigations will undoubtedly find many previously undiscovered species from submarine caves around Japan.

## Additional Information and Declarations

### Data Availability

The following information was supplied regarding data availability: The raw data (sampling locality; sampling date; condition of each specimen; museum deposition numbers, the place where the specimens are deposited) are included in the “Material and Methods” and “Material Examined” sections in the manuscript.

### New Species Registration

The following information was supplied regarding the registration of a newly described species: Publication LSID: urn:lsid:zoobank.org:pub:3019D534-3E50-49D3-AC6E-8EB5A80DD1BB; *Haimormus* (newly proposed genus name): urn:lsid:zoobank.org:act:5C5EEDB4-A9C3-406C-AA25-9D83846569AD; *Haimormus shimojiensis* (newly proposed species name): urn:lsid:zoobank.org:act:916C8C7C-5C81-43AF-9694-E7E7A372FA0C.
